# Sexual function and metabolic/hormonal changes in women using long-term hormonal and non-hormonal contraceptives: a pilot study

**DOI:** 10.1186/s12905-020-01107-1

**Published:** 2020-10-27

**Authors:** Igor Fernando de Aquino Moreira, Maria Passos Bianchini, Gabrielle Rodrigues Campos Moreira, Alessandra Maciel Almeida, Bruno Almeida Rezende

**Affiliations:** 1 Faculdade de Ciências Médicas, Instituto de Pós-graduação, Alameda Ezequiel Dias, 275 - Centro, Belo Horizonte, MG 30110-130 Brazil; 2 Hospital da Polícia Militar de Minas Gerais, Belo Horizonte, MG Brazil

**Keywords:** Female sexual dysfunction, Female sexual function index, Long-acting reversible contraception, Sexuality, Subdermal implant

## Abstract

**Background:**

Female sexual dysfunction is a common condition that negatively impacts the emotional health and quality of life of the affected individuals. Long-acting reversible contraceptives (LARCs) are becoming increasingly popular due to their effectiveness and convenience. LARCs can be hormonal (etonogestrel releasing implant—ENG and Levonorgestrel intrauterine system—LNG) or non-hormonal (copper intrauterine device—CuIUD and copper-silver intrauterine device—SIUD). There are very few studies that assess the influence on LARCS on sexual function are lacking. This study aimed to assess changes in sexual function as well as metabolic and hormonal parameters in women after implantation with LARCs.

**Methods:**

In this prospective cohort study, we assessed 80 women who visited the Military Police Hospital in Brazil for LARCs placement. The study participants were divided into 4 groups according to the type of LARC received: ENG n = 17; LNG n = 22, CuIUD n = 18 and SIUD n = 23. The four groups were evaluated twice (prior to LARC placement and approximately 3 months later) for sexual function, using the Female Sexual Function Index (FSFI) and Female Sexual Quotient (QS-F) questionnaires. Metabolic and hormonal parameters were also assessed using blood tests.

**Results:**

ENG worsened sexual function according to FSFI and QS-F, across all domains. A decrease in sex hormone-binding globulin (SHBG) between stages was observed for all groups. We observed an improvement in sexual function for non-hormonal LARCs, specially SIUD. However this improvement was not statistically significant.

**Conclusion:**

The use of non-hormonal LARCS improved sexual function. Etonogestrel implants, had a negative influence on sexual function, probably by blocking ovarian function, and thus reducing the production of androgens and estrogens.

## Background

Contraceptive techniques have progressively improved over the past few years since contraceptive pills were approved in the 1960s. The emergence of new hormonal types, dosages, and administration routes increased the effectiveness, safety and convenience of these devices [1]. Long-term reversible contraceptives (LARCs) have shown greater effectiveness and continuity compared to short-term contraceptive methods [2]. Non-hormonal LARCs include the copper intrauterine device (CuIUD) and silver intrauterine device (SIUD). These devices gradually release copper in the uterus, causing inflammatory reactions that lead to endometrial and mucus changes as well as reduced tubal motility, making the environment hostile to spermatozoids [3]. Hormonal LARCs are represented by the levonorgestrel intrauterine system (LNG) and the etonogestrel-releasing implant (ENG). LNG has a stem that releases synthetic progestin in small amounts in the uterus, causing cervical mucus thickening, endometrial atrophy, tubal motility and ciliary movement inhibition. In addition, it gets absorbed into the bloodstream and causes an ovarian function block [3]. Moreover, the device generally acts in the same way as the copper IUD—changing the uterus environment to prevent pregnancy. ENG is a subcutaneous device that systematically releases a progesterone-like synthetic substance, thereby preventing ovulation, changing the cervical mucus, and hindering the entrance of spermatozoids. This is considered the most effective contraceptive method available in the world [4].

Regardless of costs, women have preferred the use of LARCs as contraceptive methods due to their ease of use and lower incidence of side effects compared to oral contraceptives combining estrogen and progesterone [5]. The use of oral contraceptives may have a negative effect on the woman’s sexual function, which may cause the discontinuation of the method [6]. In addition, they are often associated with important metabolic changes caused by the estrogenic component of the drug, such as increased insulin resistance [7], changes in the lipid profile (increased LDL and decreased HDL), and increased risk of cardiovascular incidents [8]. Combined oral contraceptives increase thrombo-embolic events by two to four times [9, 10], and some studies suggest that these medications increase the risk of breast cancer [11].

In addition to being very effective as a contraceptive, hormonal LARCs are well known for being effective in decreasing pelvic pain and menorrhagia [2]. Non-hormonal LARCs, on the other hand, may interfere less with hormonal and metabolic parameters and, for this reason, may be preferred by some women and professionals concerned with the consequences of continuous progesterone release. These devices can reduce the fear of unwanted pregnancy, promoting a more relaxed and pleasurable sexual experience and, thus, improving the user’s sexual function [12]. This may be possible because a satisfactory sexual response and the perception of sexual pleasure during intercourse is influenced by a complex multifactorial set, involving biological, psychological, and environmental factors [12]. The way LARCs affect female sexual experiences have only been studied by researchers in the last few years [13]. Thus, further studies are needed to determine the influence of these devices on the female sexual function. Oral contraceptives have garnered more attention since the 1970s as these medications have been associated with decreased libido. This may be related to androgen metabolism, which increases the sex hormone-binding globulin (SHBG) and concomitantly decreases free testosterone and other androgens [14, 15].

Based on the association between sexuality and use of contraception, the aim of this study was to assess sexual function changes in women after the implantation of hormonal and non-hormonal LARCs, in addition to evaluating, changes in metabolic and hormonal parameters.

## Methods

### Study design

This is a prospective, cohort study conducted from October 2018 to October 2019, at the General Gynecology Clinic of the Military Police Hospital of Minas Gerais, Brazil.

### Study participants

We included women between the ages of 20–35 years who expressed the desire to use LARCs. Inclusion criteria included the desire for the use of contraception for a minimum of one-year; an active sex life (more than four relationships intercourses in the previous month); and normal oncotic cytology.

Exclusion criteria included the evidence of adrenal, cardiovascular, liver or kidney disease; uncontrolled hypothyroidism; diabetes; hyperprolactinemia; severe arterial hypertension; gynecological abnormalities (fibroids, endometriosis, congenital abnormalities, and active uterine infections); clinical history of thromboembolic disease or thrombophilia; smoking; pregnancy, lactation or abortion in the previous four months; any malignant or pre-malignant disease; history of migraines; and use of antidepressant and anticonvulsant medications.

Detailed information was provided on the duration of the study and the place of implantation. Each patient chose the desired type of LARC available, which depended on the number of samples provided by the laboratories: (i) CuIUD (Andalan Classic Cu 380®, DKT do Brasil, São Paulo, Brazil) (42 samples), (ii) SIUD (Andalan Silverflex®, DKT do Brasil, São Paulo, Brazil) (28 samples), (iii) LNG (Mirena®. Bayer SA, Bayer Oy, Turku, Finland) (32 samples), (iv) ENG (Implanon®, Schering-Plough, New Jersey, USA) (19 samples). In addition to receiving the devices free of charge, there was no cost to implant the selected method.

The sample size was calculated to compare the averages of sexual function for QS-F and FSFI instruments from four groups of LARCs. For that it would be necessary to perform a test of analysis of variance. Considering an average value of QS-F 80, standard deviation of 20, to detect a difference of 8 in the sexual function score, at least 75 individuals would be necessary in each group, with a 5% significance level and a minimum power of 80% [16]. However, due to the reduced availability of LARCs offered by the manufacturers, our sampling was less than planned. Thus, this study is characterized as a pilot study.

The participants were allocated into four groups by individual interest in the available methods, always respecting the WHO eligibility criteria for each case [17]: CuIUD group (copper intrauterine device), SIUD group (silver intrauterine device), LNG group (levonorgestrel intrauterine system), and ENG group (etonogestrel-releasing implant). The data were collected in two moments: immediately prior to LARC placement (Stage 1) and approximately 3 months later (Stage 2).

### Sexual function Instruments

Sexual function was assessed using the Female Sexual Quotient (QS-F) and Female Sexual Function Index (FSFI) questionnaires.

The QS-F is a questionnaire developed by the Program on Sexuality Studies of the Institute of Psychiatry of Hospital das Clínicas, School of Medicine of the University of São Paulo (IPq/HCFMUSP) and validated specifically for the Brazilian female population [18]. The instrument consists of ten questions with answers scored on a scale from zero to five, with zero indicating “never”, and five, “always”. It assesses the various phases of the sexual response cycle, in addition to the domains desire and sexual interest, foreplay, excitement and harmony, comfort, and orgasm and satisfaction, identifying specific dysfunctions and sexual difficulties [19]. The final score obtained is the result of the sum of the points of all questions multiplied by two, which results in a score ranging from 0 to 100. The closer to 100, the greater the sexual performance/satisfaction. Namely: 82–100 points (good to excellent), 62–80 points (fair to good), 42–60 points (unfavorable to fair), 22–40 points (bad to unfavorable), 0–20 points (null to bad). Details on the calculation of the final score can be found in the study by Abdo et al. [20].

The FSFI was developed in the United States in the 2000s, and is one of the main instruments used to assess female sexual function [21, 22].This instrument was translated into and validated for the Portuguese language [22, 23]. It consists of 19 multiple-choice questions with increasing scores ranging from 0 to 5 regarding the presence of the questioned function, and six domains (desire, excitement, vaginal lubrication, orgasm, sexual satisfaction, and pain). The total score can vary from 2 to 36 points, with values ≤ 26 indicating sexual dysfunction [22].

A questionnaire proposed by Higgins et al. [24] on the reasons why patients chose their contraceptive method was administered in the first stage of the study. This questionnaire had seven direct questions on the importance of each of the reasons presented for choosing the contraceptive method. Patients should choose between extremely important, very important, slightly important, and not at all important.

### Clinical procedures

The general medical history of all participants was investigated by the same gynecologist and they underwent gynecological examination as is routinely performed in the gynecology service of the Military Hospital of Minas Gerais. At the first consultation, the material for Pap smear was collected according to the Brazilian Guideline for cervical cancer screening of the José Alencar Gomes da Silva National Cancer Institute [25].

The study participants were assessed for clinical, metabolic, and hormonal parameters and sexual function at two different times during the study (at the initial evaluation and approximately three months after the implantation of the contraceptive device). At the first consultation, blood samples were collected for the following tests: CBC, CRP, blood glucose, glycated hemoglobin, insulinemia, prolactin, TSH, free T4, free and total testosterone, and lipid profile.

Other information relevant to the study, such as sociodemographic data, previous medical history and smoking habit were obtained from the medical records of the Military Police Hospital of Minas Gerais.

LARC implantation followed the recommendations provided by the manufacturers and the care recommended by the Brazilian Federation of Gynecology and Obstetrics (Febrasgo).

### Statistical analysis

Qualitative variables were represented as frequencies, and quantitative variables as mean ± standard deviation (median). The quantitative variables were analyzed with the Shapiro–Wilk normality test. Chi-Square test was used to evaluate the association between qualitative variables, and Kruskal–Wallis test to compare a quantitative variable among four groups. The two-way Analysis of Variance for repeated measures was used to assess the association between the types of LARC and between the stages. The association between the reasons for choosing the methods and the devices was assessed by binomial logistic regression. The analyses were performed using the free R software version 3.5.2 and p < 0.05 was considered significant.

## Results

**Fig. 1:**
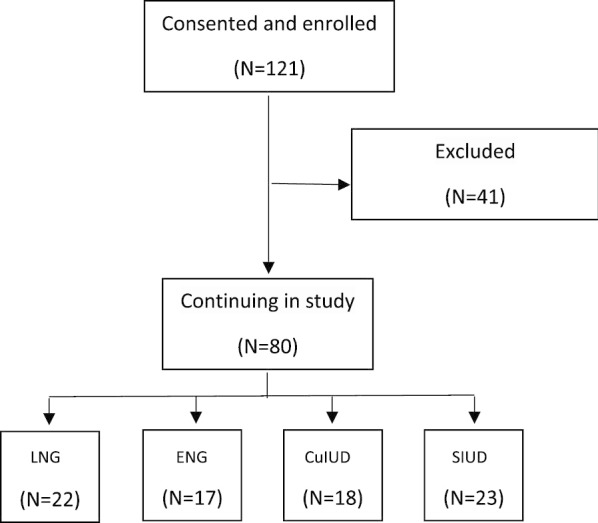
Flowchart of participant inclusion

A total of 121 women agreed to participate in the study and signed the Informed Consent Form in the first stage of the study. Of these, 41 did not attend the follow-up consultation or attended it outside the proposed period. A total of 80 patients completed the study, with the following distribution: LNG (n = 22), ENG (n = 17), CuIUD (n = 18), and SIUD (n = 23) (Fig. 1). The mean time between the first and second stage was 96 ± 10 days, the shortest period between stages was 78 days and the longest was 109 days.

The groups were homogeneously aged, with a mean age of 28.2 ± 4.3 years. Most of the patients had a college or post-graduation degree and were in a relationship of more than five years. Half of the patients reported practicing physical activity on a regular basis at least three times a week, with a minimum duration of one hour per activity. Most women had not given birth (58.4%) (Table 1). A total of 35 participants (43.7%) were using hormonal contraceptive before placing the chosen LARC with no difference among groups. Of this amount, 3 were using progestin-only contraceptive (one in LNG group and 2 in CuIUD group—data not shown). All others were using oral combined estrogen-progestin contraceptives.

**Table 1:** Socio-demographic characteristics of the study cohort

Characteristics	LNG (n = 22)	ENG (n = 17)	CuIUD (n = 18)	SIUD (n = 23)	P value	Total (n = 80)
Age (years)	28.3 ± 4 (27)	28.2 ± 4.2 (27)	27.8 ± 4.6 (27)	28.6 ± 4.3 (27)	0.962^K^	28.2 ± 4.2 (27)
"Relationship length" (years)					0.292^C^	
Less than 5	13 (59.1%)	7 (41.2%)	5 (27.8%)	13 (56.5%)		38 (47.5%)
5 to 10	5 (22.7%)	5 (29.4%)	9 (50%)	8 (34.8%)		27 (33.8%)
More than 10	4 (18.2%)	5 (29.4%)	4 (22.2%)	2 (8.7%)		15 (18.8%)
Education					0.058^C^	
High School	2 (9.1%)	3 (17.6%)	8 (44.4%)	5 (21.7%)		18 (22.5%)
College / Post-Graduation	20 (90.9%)	14 (82.4%)	10 (55.6%)	18 (78.3%)		62 (77.5%)
Physical activity (3 h / week)	11 (50%)	6 (35.3%)	6 (33.3%)	17 (73.9%)	0.053^C^	40 (50%)
Parturition					0.261^C^	
0	15 (71.4%)	9 (52.9%)	7 (41.2%)	14 (63.6%)		45 (58.4%)
1 or more	6 (28.6%)	7 (47.1%)	10 (58.8%)	8 (36.4%)		32 (41.6%)
Hormonal contraceptive use before study					0.800^C^	
None	13 (59.1%)	11 (64.7%)	9 (50%)	12 (52.2%)		45 (56.3%)
Combined estrogen-progestin/Progestin only	9 (40.9%)	6 (35.3%)	9 (50%)	11 (47.8%)		35 (43.7%)

*LNG* levonorgestrel intrauterine system, *ENG* etonogestrel-releasing implant, *CuIUD* copper intrauterine device, *SIUD* silver intrauterine device. ^C^Chi-Square test, ^K^Kruskal–Wallis test

Table 2 shows the criteria used by the patients to choose the contraceptive method. Most of them reported that it is very to extremely important that the contraceptive method is effective. In addition, they reported that it was very or extremely important not to reduce libido and not to interrupt sex (93.6% and 83.8% of the total patients in the study, respectively). The only point presenting statistically significant differences between the groups was the choice of the method without hormones—non-hormonal LARCs (CuIUD and SIUD) compared to the hormonal method ENG (P < 0.001).

**Table 2:** Reasons for choosing the contraception method

	Total (n = 80)	LNG (n = 22)	ENG (n = 17)	CuIUD (n = 18)	SIUD (n = 23)	P-value
*It's the most effective method *(quite/extremely important)	78 (97.5%)	21 (95.5%)	17 (100%)	17 (94.4%)	23 (100%)	0.567
*It doesn't reduce my libido* (quite/extremely important)	75 (93.6%)	19 (86.4%)	17 (100%)	17 (94.4%)	22 (95.7%)	0.353
*It doesn't interrupt sex* (quite/extremely important)	67 (83.8%)	16 (72.7%)	14 (82.4%)	16 (88.9%)	21 (91.3%)	0.075
*It is acceptable to my partner* (quite/extremely important)	41 (51.2%)	16 (72.7%)	12 (70.6%)	12 (66.7%)	12 (51.2%)	0.489
*It doesn't contain hormones* (quite/extremely important)	57 (71.3%)	11 (50%)	7^*‡^ (41.2%)	17^‡^ (94.4%)	23^*^ (100%)	** < 0.001**
*It's recommended by my friends* (quite/extremely important)	39 (48.8%)	13 (59.1%)	7 (41.2%)	9 (50%)	10 (45.5%)	0.711
*It's in line with my religious beliefs* (quite/extremely important)	22 (30.4%)	9 (40.9%)	4 (23.5%)	2 (11.1%)	7 (30.4%)	0.145

*LNG* levonorgestrel intrauterine system, *ENG* etonogestrel-releasing implant, *CuIUD* copper intrauterine device, *SIUD* silver intrauterine device. The p-values refer to the binary logistic model, and the symbols ‡ and * indicate the pairs in which they differ

**Fig. 2:**
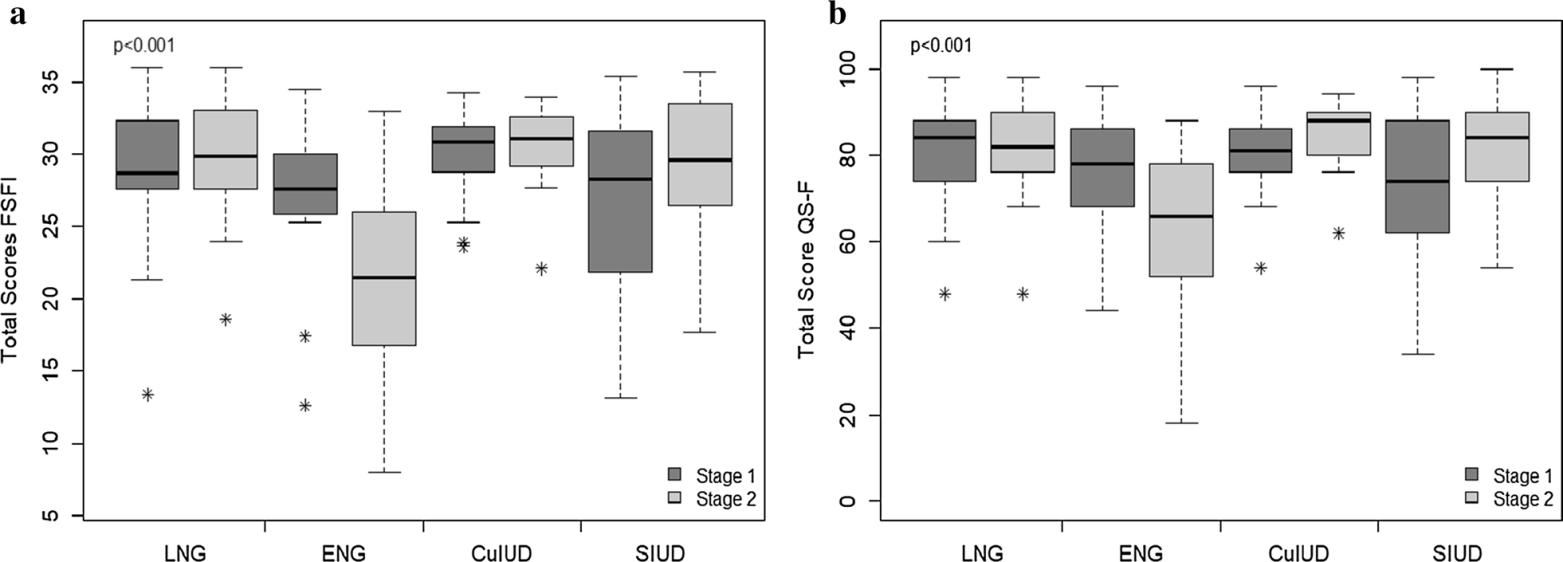
Box diagrams for the total scores of the Female Sexual Function Index (FSFI—(**a**) and Female Sexual Quotient (QS–F—B) instruments for the different types of LARCs. The p-values refer to Two-way Analysis of Variance

As for the hormonal and metabolic parameters evaluated in both stages of the study (Table 3), all LARC groups showed decreased levels of SHBG following implantation (P < 0.001). However, for the CuIUD group, this difference was not significant in post-hoc test. Following implantation, the other metabolic parameters evaluated showed no significant changes between the two stages of the study for the groups studied. When comparing sexual function between the two stages of the study, using both the FSFI (Table 4) and QS-F (Table 5) it was not observed significant changes for the 4 types of LARCs evaluated (p-value^2^ > 0,05). Comparing difference between LARCs, ENG users presented worse results in all domains assessed by both instruments (p-value^1^ < 0.001) for total FSFI and QS-F scores (Fig. 2). For non-hormonal LARCs, the data suggest an improvement in sexual function. However, the increase observed in individual domains or total scores for these devices showed no statistical significance (Tables 4 and 5).

**Table 3:** Hormonal and metabolic parameters at the different stages of the study for the 4 types of LARCs evaluated

Parameter	LNG	ENG	CuID	SIUD	P value^1^	P value^2^
SHBG (nmol/L)					0.403	** < 0.001**
Stage 1	97.5 ± 79* (68.8)	112.5 ± 73.7^†^ (84.4)	71.4 ± 51.2 (54.9)	100.4 ± 79.2^£^ (77)		
Stage 2	51.7 ± 17.1* (52.6)	61.8 ± 18^†^ (63.5)	46.1 ± 13.4 (47.3)	38.2 ± 16.1^£^ (32.7)		
Total testosterone (mg/dL)					0.301	0.141
Stage 1	31.3 ± 21.2 (24.4)	26 ± 15.8 (21.2)	27.4 ± 17.1 (22.1)	25.3 ± 9.6 (24)		
Stage 2	33.5 ± 8.7 (32)	26.3 ± 7 (27.3)	32.3 ± 8.8 (31.6)	32.7 ± 8.8 (34.8)		
Free testosterone (mg/dL)					0.519	0.941
Stage 1	0.4 ± 0.5 (0.2)	0.3 ± 0.3 (0.2)	1 ± 2.5 (0.3)	1.1 ± 3.9 (0.2)		
Stage 2	0.3 ± 0.3 (0.3)	0.3 ± 0.1 (0.3)	1.8 ± 5.8 (0.3)	0.4 ± 0.2 (0.3)		
TSH (mU/L)					0.833	0.327
Stage 1	2.1 ± 1 (1.8)	2 ± 1 (1.6)	2.1 ± 1.7 (1.5)	2.5 ± 1.9 (2.1)		
Stage 2	2.1 ± 0.7 (2.2)	2.2 ± 0.8 (2.1)	1.9 ± 1 (1.6)	1.7 ± 0.7 (1.7)		
Free T4 (ng/dL)					0.577	0.249
Stage 1	0.9 ± 0.2 (0.9)	0.9 ± 0.3 (0.9)	1 ± 0.3 (1)	1 ± 0.2 (1)		
Stage 2	0.9 ± 0.2 (0.9)	1.1 ± 0.2 (1.1)	1.1 ± 0.2 (1.1)	1 ± 0.2 (1)		
Prolactin (ng/mL)					0.458	0.100
Stage 1	19.3 ± 18.8 (12.1)	20.5 ± 15.1 (13.9)	17.9 ± 13.6 (12.6)	14.9 ± 9.6 (10.9)		
Stage 2	10.8 ± 5.1 (9.3)	12.5 ± 5.6 (11.2)	11.6 ± 5.8 (9.3)	10.1 ± 3.2 (9.4)		
Insulin (mU/L)					0.234	0.534
Stage 1	7.4 ± 3.1 (6.6)	6.5 ± 4.9 (5.4)	6.7 ± 3.8 (6)	6.6 ± 5.1 (5.3)		
Stage 2	6.3 ± 1.7 (6.4)	6.6 ± 2.2 (5.7)	6.9 ± 3.1 (6.2)	6.2 ± 3 (5.3)		
HDL (mg/dL)					0.058	0.922
Stage 1	59 ± 16.6 (54)	55 ± 12.4 (55)	58.1 ± 15.9 (55)	62.1 ± 16.2 (59)		
Stage 2	60.8 ± 11.7 (57)	53.1 ± 10.8 (52)	60.5 ± 11 (55)	60.8 ± 11.3 (55)		
Triglycerides (mg/dL)					0.398	0.471
Stage 1	84.5 ± 40.7 (71.5)	98.5 ± 47.8 (100)	72.5 ± 36.6 (66.5)	85.9 ± 38.2 (70)		
Stage 2	83.5 ± 23.5 (78)	86.1 ± 27.5 (85)	75.8 ± 24.8 (73.5)	78.8 ± 26.6 (71)		
LDL(mg/dL)					0.314	0.495
Stage 1	97.7 ± 22.5 (100.5)	93.6 ± 30.4 (88)	105.3 ± 26.7 (100.5)	101.1 ± 21.7 (100)		
Stage 2	103.6 ± 21.5 (103)	93.1 ± 22.5 (88)	107.5 ± 24 (101)	103.1 ± 16.2 (105)		
Fasting glycemia (mg/dL)					0.827	0.456
Stage 1	82 ± 6.7 (82.5)	80.6 ± 6.3 (83)	79.3 ± 9 (79.5)	80.8 ± 7.5 (81.5)		
Stage 2	80.4 ± 4.1 (81)	78.9 ± 9.6 (82)	80.1 ± 5.2 (81)	80.1 ± 4.4 (81)		
Glycatedhemoglobin (%)					0.359	0.477
Stage 1	5.3 ± 0.4 (5.3)	5.1 ± 0.3 (5.1)	5.4 ± 0.4 (5.5)	5.3 ± 0.3 (5.2)		
Stage 2	5.2 ± 0.4 (5.2)	5.2 ± 0.2 (5.3)	5.2 ± 0.3 (5.2)	5.3 ± 0.3 (5.2)		
Hemoglobin (g/dL)					0.638	0.224
Stage 1	13.4 ± 0.8 (13.6)	13.4 ± 1.3 (13.2)	13.7 ± 0.8 (13.7)	13.4 ± 0.8 (13.5)		
Stage 2	13.8 ± 0.6 (14.1)	13.7 ± 0.8 (13.5)	13.2 ± 0.6 (13.2)	15 ± 7 (13.2)		

*LNG* levonorgestrel intrauterine system, *ENG* etonogestrel-releasing implant, *CuIUD* copper intrauterine device, *SIUD* silver intrauterine device. P value ^1^refers to comparison between the LARC types and p value ^2^refers to comparison between stages. Symbols *,^†^, ^£^indicates pairs with significative differences in post-hoc test. Data presented as mean ± SD (median)

**Table 4:** Comparison of domain scores for Female Sexual Function Index (FSFI) questionnaire in the different stages of the study for the 4 types of LARCs evaluated

Domain	LNG	ENG	CuID	SIUD	P value^1^	P value^2^
Desire					<* 0.001*	0.165
Stage 1	4 ± 0.9 (3.9)	3.5 ± 1.1 (3.6)	3.6 ± 0.9 (3.6)	3.7 ± 1.4 (3.6)		
Stage 2	4.3 ± 0.9* (4.2)	2.6 ± 0.9*^†£^ (2.4)	4.2 ± 0.8^†^ (4.2)	4.4 ± 1.2^£^ (4.8)		
Arousal					<* 0.001*	0.943
Stage 1	4.8 ± 0.9 (4.8)	4.4 ± 0.9 (4.8)	4.8 ± 0.8 (5.1)	4.3 ± 1.2 (4.5)		
Stage 2	4.8 ± 0.9* (4.8)	3.4 ± 1.3*^†£^ (3.6)	5 ± 0.5^†^ (5.1)	4.9 ± 0.8^£^ (4.8)		
Lubrification					<* 0.001*	0.928
Stage 1	5 ± 0.9 (4.8)	4.7 ± 1.2 (4.8)	5.7 ± 0.5 (5.9)	4.6 ± 1.3 (4.8)		
Stage 2	5.2 ± 0.8* (5.4)	3.5 ± 1.4 *^†£^ (3.9)	5.7 ± 0.5^†^ (6)	5.2 ± 1^£^ (5.7)		
Orgasm					*0.002*	0.460
Stage 1	4.6 ± 1.4 (4.8)	4.3 ± 1.2 (4.4)	5 ± 0.8 (5.2)	4.2 ± 1.6 (4.4)		
Stage 2	4.6 ± 1.4* (4.8)	3.3 ± 1.4*^†£^ (3.2)	4.9 ± 0.8^†^ (5.2)	4. 5 ± 1.5^£^ (4.8)		
Satisfaction					*0.004*	0.444
Stage 1	5 ± 1.2 (5.4)	5.2 ± 0.9 (5.2)	5.4 ± 0.8 (5.6)	4.7 ± 1.2 (4.8)		
Stage 2	5.2 ± 0.9* (5.4)	4.1 ± 1.3*^†£^ (4.4)	5.2 ± 0.7^†^ (5.2)	5 ± 0.9^£^ (5.2)		
Pain					*0.001*	0.993
Stage 1	5.5 ± 0.7 (6)	5.1 ± 1.3 (6)	5.4 ± 1 (6)	4.9 ± 1.4 (5.2)		
Stage 2	5.5 ± 1* (6)	4.3 ± 1.6*^†£^ (4.8)	5.8 ± 0.5^†^ (6)	5.3 ± 1^£^ (6)		
Total Score					* < 0.001*	0.958
Stage 1	28.9 ± 4.8 (28.7)	27.1 ± 5.3 (27.6)	30 ± 3 (30.8)	26.3 ± 6.7 (28.3)		
Stage 2	29.6 ± 4* (29.8)	21.2 ± 6.9*^†£^ (21.5)	30.7 ± 2.9^†^ (31.1)	29.3 ± 4.8^£^ (29.6)		

*LNG* levonorgestrel intrauterine system, *ENG* etonogestrel-releasing implant, *CuIUD* copper intrauterine device, *SIUD* silver intrauterine device. P value ^1^refers to comparison between the LARC types and p value ^2^refers to comparison between stages. Symbols *,^†^, ^£^indicates pairs with significative differences in post-hoc test. Data presented as mean ± SD (median)

**Table 5:** Comparison of domain scores for Female Sexual Quotient (QS-F) questionnaire in the different stages of the study for the 4 types of LARCs evaluated

Parameter	LNG	ENG	CuID	SIUD	P value^1^	P value^2^
Desire					*0.002*	0.334
Stage 1	21.3 ± 4 (22)	20.6 ± 5.2 (20)	21.3 ± 3.8 (22)	19.7 ± 8.1 (22)		
Stage 2	22.4 ± 4.1* (22)	16.5 ± 6.5*^†£^ (16)	23.2 ± 3.4^†^ (23)	23.1 ± 4.6^£^ (24)		
Preliminary					*0.001*	0.053
Stage 1	9.6 ± 0.8 (10)	9.2 ± 1.2 (10)	9.2 ± 1.6 (10)	9 ± 1.5 (10)		
Stage 2	9.1 ± 1.4* (10)	7.3 ± 2.6*^†£^ (6)	9.4 ± 0.9^†^ (10)	9.1 ± 1.3^£^ (10)		
Arousal					< *0.001*	0.534
Stage 1	17.1 ± 2.5 (18)	15.4 ± 3.8 (16)	18 ± 2.3 (18)	16 ± 4.2 (18)		
Stage 2	17.1 ± 2.4 * (16)	12.1 ± 4.8*^†£^ (14)	18.1 ± 2.6^†^ (19)	17.2 ± 2.9^£^ (18)		
Comfort					*0.045*	0.069
Stage 1	15.9 ± 2.9 (16)	15.8 ± 3.4 (18)	16.6 ± 3.4 (17)	14.2 ± 4.7 (16)		
Stage 2	16.8 ± 3.1 (18)	14.4 ± 4.8^†^ (16)	18.2 ± 1.7^†^ (18)	16.8 ± 3.5 (18)		
Orgasm & Satisfaction					< *0.001*	0.471
Stage 1	16.5 ± 4.4 (18)	14.5 ± 3.8 (16)	15.4 ± 3.5 (16)	14 ± 4.4 (14)		
Stage 2	16.4 ± 4.2* (18)	10.6 ± 4.7*^†£^ (10)	16.1 ± 2.8^†^ (16)	14.9 ± 3.9^£^ (16)		
Total score					< *0.001*	0.791
Stage 1	80.4 ± 11.5 (84)	75.4 ± 14 (78)	80.6 ± 9.8 (81)	73 ± 18.4 (74)		
Stage 2	81.8 ± 11.5* (82)	60.8 ± 21.1*^†£^ (66)	85.1 ± 8.2^†^ (88)	81.1 ± 13.2^£^ (84)		

*LNG* levonorgestrel intrauterine system, *ENG* etonogestrel-releasing implant, *CuIUD* copper intrauterine device, *SIUD* silver intrauterine device. P value ^1^refers to comparison between the LARC types and p value ^2^refers to comparison between stages. Symbols *,†,£indicates pairs with significative differences in post-hoc test. Data presented as mean ± SD (median)

## Discussion

The main finding of this study is the worsening of sexual function in patients after ENG implantation involving the use of two instruments of sexual function. The QS-F was developed specifically for the Brazilian female population. However, the FSFI is an instrument consolidated internationally and the use of both tools increased the study reliability, since both instruments showed the same outcome.

Most women evaluated in this study consider it very or extremely important that the contraceptive method chosen does not change their libido or interrupt the sexual act. This, together with the effectiveness of the method, were the most important reasons for choosing the contraceptive method in the groups evaluated, reflecting the great importance given by patients to maintain sexual function. Assessing female sexual function is important, as it interferes with quality of life and is generally associated with general health issues [26]. Abdo et al. [20] show that 49% of Brazilian women have some degree of sexual dysfunction, including decreased libido, dyspareunia, or dysfunctional orgasm. This study did not assess the prevalence of sexual dysfunction for the methods evaluated, but rather the sexual function changes resulting from LARC implantation.

Intrauterine devices that combine silver and copper began to be used in the USA in the 1970s, showing lower pregnancy rates when compared to devices only containing copper [27].The addition of silver to the device aimed at preventing corrosion [28], with copper release seeming to remain unchanged when silver is added [28, 29]. Although the combined use of copper and silver is already well established in many countries, in Brazil, this device started to be marketed in 2016 under the name Andalan Silverflex®. To date, no study was found comparing sexual function changes in SIUD and classic devices users without the incorporation of silver. Our study suggest that non-hormonal devices can improve sexual function when compared to hormonal devices, and SIUD seems to show even more substantial improvement when compared to CuIUD in both instruments used.

Despite studies showing that the use of contraceptives increases libido in women with decreasing concerns about an unwanted pregnancy [30], this study shows a worsened sexual function, including libido, with ENG. The literature presents conflicting results regarding the influence of ENG on sexual function. As described by Bason (2001), the female sexual response is complex, because it is influenced by a multifactorial factors including biological, psychological and sociocultural [31]. The psychological factor was analyzed in a study that demonstrated decreased vitality and emotional function in patients in the first three months of ENG use [32]. However, this study reported no worsening of sexual function in the group studied. The study by Bozalis et al. (2016), based on a large database (CHOICE), showed that ENG users reported the loss of sexual interest more frequently when compared to CuIUD users [9]. Two studies evaluating the side effects of ENG use in women followed up for about two years show a prevalence of 2.5% and 1.6% decreased libido, respectively [33, 34]. Decreased libido was associated not only with ENG, but also with depot medroxyprogesterone acetate (DMPA) injections, which may be related to systemic progestin release [9, 35]. Estrogens have a fundamental role in female sexuality and their administration can be a recommended treatment for low libido and hypoactive sexual desire disorder [36]. Systemic progestins can suppress ovarian function and consequently decrease the natural production of estrogen, resulting in loss of sexual desire [9]. In addition, ENG is the device with the highest rate of discontinuation among LARCs users [37]. In the present study, five (29%) of the 17 ENG users discontinued the method within six months after the second stage of the study (data not shown). Some studies associated the discontinuation of LARCs with sexual function changes [12, 38, 39], suggesting a negative effect of ENG on sexual function.

This study also correlated metabolic and hormonal parameters with sexual function findings. Total testosterone levels slighted increased in non-hormonal LARCs users and remained constant in hormonal LARCs users. These results may be related to the fact that some women in this study were using combined oral contraceptive methods before starting LARC implantation, which is known to reduce total testosterone [40, 41]. Since SIUD and CuIUD contain no hormones, their use would make it possible to re-establish the androgen production axis, whereas, for hormonal LARCs, this re-establishment could occur partially or not occur. The decrease in SHBG for all groups in this study may also be due to the use of combined oral contraceptives prior to LARC implantation. The combined oral contraceptives agents are known increase the hepatic production of SHBG [40, 41]. Many studies suggest that this is one of the reasons why the use of combined oral contraceptives reduce libido, since excess SBGH would decrease the free testosterone fraction in women, directly affecting sexual function [14, 15]. After stop using combined oral contraceptives a decrease in SHBG would be expected. In addition, the fact that the higher levels of total testosterone observed in users of non-hormonal LARCs could also be related to improved sexual function in these groups cannot be excluded.

The slightly non-significant increase in hemoglobin levels observed in women using the LNG and ENG progestin hormonal methods in this study corroborates other studies [42, 43]. Likewise, reduced hemoglobin levels in CuIUD were already analyzed in many studies [44, 45]. The SIUD group, however, showed hemoglobin increase, which may mean a better SIUD response compared to CuIUD regarding blood loss. These data could be related to the smaller copper surface covering the SIUD, generating less inflammatory reaction and, therefore, less bleeding during the menstrual period [46].

Even not being statistically significant, this present study observed decreased prolactin levels in all groups evaluated. Once again, the use of combined oral contraceptives may have influenced these results. Prolactin levels has been studied for non-hormonal LARCs since the 1970s, and these studies report that these devices cannot influence the plasma levels of this hormone [47, 48]. However, ethinyl estradiol and levonorgestrel oral contraceptives presented a considerably increased prolactin and macroprolactin plasma levels [49, 50]. Thus, the discontinuation of oral contraceptives through the implantation of LARCs could re-establish baseline levels. Another factor that could interfere with serum prolactin levels is pulsatility, since these levels vary within the normal range throughout the ovulatory cycle, reaching a maximum of 20 ng/ml near ovulation [51]. Slightly increased T4 baseline levels were observed for LNG, but TSH levels remained unchanged in this group, suggesting that this change may be unimportant. Lipid parameters seemed to be stable after LARC implantation, which corroborates the literature [52].

## Conclusion

This study assessed the influence of four long-term reversible contraceptive methods available in the market on female sexual response. The use of etonogestrel implants was associated with a worsened sexual response when assessed by the FSFI and QS-F instruments. Blocked ovarian function and a probable reduction in androgen and estrogen production may explain this result. The non-hormonal devices tend to show an improvement in sexual function.

## Study limitations

This study included women in use of oral contraceptives prior to LARC implantation. The previous use of these agents is believed to have influenced the study results, mainly the metabolic parameters. However, the percentage of oral contraceptives users was quite homogeneous among the groups evaluated. Another study limitation includes a small sample size. New studies with a larger sample are needed to confirm the findings of this study.

## Data Availability

The dataset analyzed during the current study is available from the corresponding author on reasonable request.

## Abbreviations

CuIUD: Copper intrauterine device
ENG: Etonogestrel releasing implant
FSFI: Female Sexual Function Index
HDL: High density lipoprotein
LARCs: Long-acting reversible contraceptives
LDL: Low density lipoprotein
LNG: Levonorgestrel intrauterine system
QS-F: Female sexual quotient
SIUD: Copper–silver intrauterine device
SHBG: Sex hormone-binding globulin
T4: Thyroxin WHO: World Health Organization
